# Climate mitigation and intensified forest management in Norway: To what extent are surface waters safeguarded?

**DOI:** 10.1007/s13280-020-01357-1

**Published:** 2020-09-12

**Authors:** Frode Sundnes, Marianne Karlsson, Froukje Maria Platjouw, Nicholas Clarke, Øyvind Kaste, Salar Valinia

**Affiliations:** 1 Norwegian Institute for Water Research (NIVA), Gaustadallèen 21, 0349 Oslo, Norway; 2 Norwegian Institute of Bioeconomy Research (NIBIO), Høgskoleveien 8, 1433 Ås, Norway; 3 University of Agder, Centre for Coastal Research, Pb 422, 4604 Kristiansand, Norway

**Keywords:** Afforestation, Climate mitigation, Fertilisation, Forest management, Intensification, Surface waters

## Abstract

While the role of forestry in mitigating climate change is increasingly subject to political commitment, other areas, such as water protection, may be at risk. In this study, we ask whether surface waters are sufficiently safeguarded in relation to the 2015 launch of a series of measures to intensify forest management for mitigation of climate change in Norway. First, we assess how impacts on water are accounted for in existing regulations for sustainable forestry. Secondly, we provide an overview of the impacts of forestry on water quality relevant to three support schemes: afforestation on new areas, increased stocking density in existing forests, and forest fertilisation. Lastly, we assess the uncertainties that exist with regard to surface waters in the implementation of these measures. We find that the safeguards in place are adequate to protect water resources at the point of initiation, but there is a large degree of uncertainty as to the long-term effect of these mitigation measures.

## Introduction

Based on the 2012 white paper *the Norwegian Climate Policy*, the Norwegian parliament came to an agreement (“Klimaforliket”) that states Norway’s ambitions for meeting international obligations on emission reductions (KLD 2012). While Norway through its 2007 “climate and forest initiative” has shown strong commitment to fight deforestation in tropical forests, the new agreement of 2012 emphasised the forest’s carbon sink capacity in Norway. One of the stated goals was to maintain or increase the forest carbon stock through active, sustainable forest policies, with particular reference to new actions to intensify the forest industry. This reflected a new direction of Norwegian climate policy where two-thirds of emission cuts are now to be made nationally. These national commitments are reiterated in the 2015 white paper *New emission commitment for Norway for 2030*—*towards joint fulfilment with the EU* (KLD 2015). The new actions introduced here included afforestation on new areas, increased stocking density, and fertilisation of forests. The details of these measures are presented below.

We view these climate mitigation measures as forms of *intensification* of the forestry sector as they are put in place with the aim of increasing the biomass produced per unit forest area. Although the measures represent a change towards intensification, it should be noted that the Norwegian forest industry is a far cry from being labelled as an intensive industry. A large proportion of forest areas in Norway are not actively managed due to topographical conditions and lack of accessibility (Ring et al. 2017). Further, due to the fragmented nature of Norwegian forest holdings, both topographically and in terms of ownership structure, the Norwegian forestry sector is small compared to that of its neighbouring countries such as Sweden and Finland (Table 1).

**Table 1:** Overview of forest production in Fennoscandia

	Norway	Sweden	Finland
Forest land area (Mha)^1) 2)^	12.1	28.1	22.2
Proportion of forest land of total land area (%)^a,b^	39.8	68.4	73.1
Production forest, incl. multiple use forest (Mha)^c^	11.5	23.5	18.9
Production forest, excl. multiple use forest (Mha)^c^	6.6	19.7	n/a
Proportion production forest (incl. multiple use forest) to forest land area (%)	95.0	83.6	85.1
Proportion production forest (excl. multiple use forest) to forest land area (%)	54.5	70.1	n/a
Growth rates, total growing stock volumes, 1995–2015^c^	0.47	0.19	0.24

^a^Ring, Johansson et al. (2017)

^b^FAO (2015)

^c^FAO 2015 Country reports

Intensified forest management through increased productivity may involve significant trade-offs as it can compromise other important ecosystem services, such as harvesting of non-timber product, recreation, cultural heritage, pasture, biodiversity, and water quality (Framstad et al. 2009; Laudon 2011; Nordin et al. 2011; Sandström et al. 2011; Duncker et al. 2012).

In this paper, we investigate how and in which ways effects on surface waters are considered in the implementation of the climate mitigation measures in the forest sector in Norway. We take as a starting point in this paper that measures that intensify forestry may also lead to impairment of surface waters in Norway. The severity of these impacts is, however, dependent on what type of forest harvesting method is used and on what temporal resolution the negative effects are evaluated (Futter et al. 2019). Also, forest owners and operators in Norway are guided by a set of legal requirements and regulations that *inter alia* address the possible impacts of forestry operations on surface waters.

In addition to the three above-mentioned measures, general approaches to reduced deforestation have also been flagged as important for Norway’s long-term approach to reduce carbon emission (KLD 2015). Further, conservation of standing forests is also of great relevance for the discussions on climate mitigation within the Norwegian forestry sector (see Flugsrud et al. 2016). Since our focus is on measures that lead to intensification of the forestry sector, we have chosen not to include conservation and reduced deforestation in our assessment.

Our review of the impacts of climate mitigation measures in forestry on surface waters in Norway is based on literature from Norway and neighbouring Fennoscandian countries on impacts on surface water relevant to these measures. We also reviewed national and international laws, regulations, guidelines and policy documents relevant for forestry in Norway, including documentation on the reception and implementation of these measures.

The following section provides an overview of the general legal framework applicable to forestry and intensification measures. This will be followed by an assessment of the three different intensification measures and their possible effects on the quality of surface waters.

## The legal framework for forestry intensification

Sustainable forestry is regulated through international policies, criteria and indicators, as well as national regulations, principles and standards. On the international policy arena we have over the last decade seen an increasing integration of *water* and *forestry* issues, concurrently with an increasing focus on the forestry sector’s potential contribution to climate mitigation. The close interrelation between forests and water was recognised in 2007 by the European *Warsaw Resolution 2 on Forests and Water*, emphasising the role of forests and forest management for biodiversity of water ecosystems and for protection of water quality (Forest Europe 2007). The more recent 2030 Agenda for Sustainable Development also acknowledges the interlinkages between water resources and sustainable forest management (UN-GA 2015). At its core are the Sustainable Development Goals promoting sustainable management of forests while at the same time calling for sustainable use and protection of freshwater ecosystems.

The EU Water Framework Directive (WFD) that was adopted in 2000 marked a significant change in European water governance, with its aim of achieving “Good Ecological Status” of surface waters and coastal waters by 2021 (WFD 2000). One of the innovations was the requirement for EU member and associated states to establish river basin districts based on geographical and hydrological criteria instead of administrative or political boundaries (Squintani and van Rijswick 2016). Although not a member state of the EU, Norway has in accordance with the European Economic Area agreement fully implemented the WFD, with a delay of one planning cycle to the EU member states. Despite its ambitious nature, this directive carries no reference to forestry and forest management. A consequence of this blind spot in the directive is that anthropogenic impacts of forestry operations on water bodies are difficult to include in planning and implementation of relevant measures to improve water quality (Futter et al. 2011; Valinia et al. 2012). The 2013 EU Forest Strategy, however, stipulates that the EU needs a policy framework that coordinates and ensures coherence of forest-related policies and allows synergies with other sectors that influence forest management. The Forest Strategy explicitly recommends to integrate sustainable forestry practices in the Programme of Measures of River Basin Management Plans under the WFD (Squintani and van Rijswick 2016, p. 4).

In tandem with this increased focus on the interlinkages between sustainable forestry and water quality, there has been a growing interest, commitment and investment in forests as carbon sinks. The *Warsaw Resolution,* for example, states clear ambitions for developing appropriate policies and strategies for managing forests and water resources sustainably to adapt to climate change and contribute to its mitigation (Forest Europe 2007; Squintani and van Rijswick 2016). Not only has the climate mitigation potential of tropical rainforest been lifted to the international policy agenda; the role of forest in general, including boreal forests, has also received increased attention. The commitment under the Kyoto Protocol for signatory states to ensure by 2020 that greenhouse gas emissions from land use are compensated by an equivalent absorption of CO_2_ made possible by additional sector-wise actions, illustrates this development well. Furthermore, in the Land Use, Land-Use Change and Forestry (LULUCF) sector, the EU and Member States have committed to maintaining and enhancing forest cover to ensure soil protection, water quality and quantity regulations (EC 2013, p. 10). For Norway, the ambitions are that 2/3 of emission cuts are to be made nationally, and carbon capture and sequestration of Norwegian forests equals half of the national total emissions (KLD 2015).

Notwithstanding these developments, an increasing targeting of forestry in climate change mitigation initiatives, and an increased focus on the water-forestry nexus, impacts on water quality and aquatic ecosystems from forestry intensification measures have been given less attention.

## Forestry regulations in Norway

In Norway, measures within forestry are regulated by the 2005 Forestry Act, which in general terms sets responsibilities and restrictions for the promotion of sustainable management of forest resources. The existing legal framework places a large responsibility on the forest owners, who shall ensure that all activities in the forests are carried out in compliance with statutes and regulations. A forest management plan is required, and it should include forest inventories, listing forest and environmental resources and values on the property, along with a plan for management of these. The inventories of environmental values shall also be publicly available. Beyond this, the forest owner has large degrees of freedom to manage the forest in relation to her/his own objectives (LMD 2005, §4). Hence, the forest owner has been given a large share of responsibility to ensure that measures and activities are carried out in a sustainable manner. In practice though, the strong involvement of forest owners’ associations in the practical forestry operations implies that single forest owners may experience this responsibility as a shared rather than an individual responsibility.

The Nature Diversity Act of 2009 aims to protect biological, geological and landscape diversity and ecological processes through conservation and sustainable use. The Act also applies to forestry. Of particular importance for the forest owners is the general duty of care; that any person, including forest owners, shall act with care and take all reasonable steps to avoid causing damage to biological, geological and landscape diversity (KLD 2009).

There are also avenues for protection of surface waters in the 2008 Planning and Building Act, through which the municipalities can establish zoning plans specifying use, conservation and design of land and physical surroundings, such as designating use and conservation of water resources (KMD 2008, art. 12.6). If there is a concern for the quality of water resources, the Environmental Protection Agency has the opportunity to propose the area as a protected area or a protected landscape (KLD 2009), which will affect the degree of activity allowed in the area and can require changes to forest management practice.

The legal framework in Norway is considered to contain a relatively high degree of prescriptiveness compared to other Nordic countries (Ring et al. 2017). This means, that despite Norway’s relatively low proportion of public forest, Norwegian legislation is procedurally prescriptive in nature by emphasis on the importance of conserving ecological values and maintaining integrity of ecological systems. This is evident in the more detailed regulations on protective measures given in the 2006 Regulation on Sustainable Forestry, specifying that forestry operations should adhere to the requirements of the national PEFC standard (LMD 2006).

The Norwegian PEFC standard, also known as the Norwegian adaptation of the Programme for the Endorsement of Forest Certification, is the forest industry’s own standard and certification scheme that sets criteria for sustainable forest management. Currently, 75% of Norway’s forest land is certified under the PEFC standard (Ring et al. 2017). In practice, though, close to all forest produce on the Norwegian market falls under the PEFC standard, as the remaining 25% forest areas are not under active production.^1^ Other Nordic countries have their own national Forest Stewardship Council (FSC) standards for certification running in parallel with their PEFC standard. The Norwegian FSC standard was discontinued in 2010, together with the collapse of the Living Forests initiative, although negotiations for a new standard are underway. Regardless of the current absence of a national standard, some 3% of Norway’s forest areas are certified by the international FSC standard.

The PEFC standard emphasises that the forests must be managed sustainably in a manner that gives financial returns to the forest owner, adds value at local and national levels, and makes a positive climate contribution, while also safeguarding outdoor recreation and environmental values (PEFC 2015, req. 1). The PEFC standard specifically requires the incorporation of a long-term perspective in forestry planning, and the forest’s contribution in the absorption and storage of carbon (PEFC 2015, req. 3). The PEFC Standard aims at safeguarding biodiversity and water resources by guaranteeing the water quality in lakes and waterways and creating habitats for species which naturally live in or near to waterways. The Norwegian PEFC Standard requires a variety of protection zones, such as the 10-15 m buffer zones, and vegetation belts around lakes, rivers and streams (PEFC 2015, req. 24).

There is criticism raised against the forest industry’s standards in Fennoscandian context, as a higher degree of voluntary certification is not necessarily accompanied by positive environmental impacts (Johansson and Lidestav 2011). Kuuluvainen et al. (2019) argue that in Finland the PEFC standard lacks scientific credibility in safeguarding biodiversity when it comes to retention practices. Hence, the protective measures stipulated by the PEFC standard are not necessarily sufficient in safeguarding environmental values. However, the PEFC standard provides guidelines at a level so detailed that we consider that the requirements of the legal framework in Norway to a large extent are fulfilled if a forestry measure or activity is carried out in accordance with the PEFC standard, such as the duty of care regulation under the Nature Diversity Act.

In light of the legal framework we will in the following section explore more specifically the effects on surface waters under the three different intensification measures.

## Exploring the impacts of intensified forestry on surface water quality

In Norway, there is a lack of long-term empirical studies to assess what potential effects intensified forest management has on water quality and quantity, although a few studies have been conducted historically (e.g. Haveraaen 1981) and more recent studies have focused on soil solution parameters (Clarke et al. 2018a). Studies from other boreal and northern temperate countries might also be applied to Norway, although care might need to be taken because of differences in forest management between countries even when the natural conditions are similar. In a recent study Futter et al. (2019) made an overview assessment of forest management effects on surface water quality using a modified version of the DWARF framework (Futter et al. 2016). The methodology was adapted to the three main climate mitigation measures and Norwegian environmental conditions. Potential effects on surface waters were here assessed on three temporal scales: 1 year after harvest, 10 years after harvest and 100 years after harvest (Futter et al. 2019). It is important to highlight that the measures proposed by the Norwegian government might not give a noticeable environmental effect directly after implementation, but effects might occur many years later and upon forest harvesting. A challenge in this respect is a general lack of long-term (one rotation or more) field experiments, making it hard to test long-term modelling empirically.

Forest management impacts surface waters in Norway, but the severity of the impact is dependent on what type of forest harvest method is used and on what temporal resolution the negative effects are evaluated (Futter et al. 2019). Any evaluation of environmental consequences of a measure must consider the whole rotation period from initial planting to harvest. The most visible and long-lasting effects of forestry occur at final harvest (Akselsson et al. 2007; Zetterberg et al. 2016), and usually not during afforestation, replanting or fertilisation, although measures can also have immediate, but short-term consequences for water quality (Löfgren et al. 2016).

We will in the following assess the three climate mitigation measures that Norway has launched for the forestry sector. By assessing how these measures have been received by the forest actors, and the extent to which water is safeguarded, we will point at possible weaknesses and uncertainties in the way these schemes have been devised and put into practice.

### Afforestation on new areas

Government support to afforestation on new areas was introduced in 2015 as a three-year pilot project in the three counties Nord-Trøndelag and Rogaland (from 2015), and Nordland (from 2016). These three pilot regions are chosen to represent three different climatic regions of coastal Norway. The initial scheme was set up to support planting of forest on new areas with NOK 15 mill for the first of three pilot years, and the measure is a continuation of earlier and ongoing attempts at facilitating afforestation in coastal regions of Norway. In a report on coastal forestry from 2008 the potential for afforestation is shown by recommending that 500 000 ha in the coastal region of Norway is afforested within the next 50 years (Øyen 2008). A more realistic, yet ambitious, estimate indicates that 2 500 ha of new forests could be planted within the next 20 years at a national level (Haugland et al. 2013). The reception of this scheme was slow, and considerable effort by regional authorities has been put into convincing forest owners of potential economic and climate gains, and also identifying suitable areas for planting. During the 5-year pilot a total of 628 ha was afforested in these three counties, spread across 189 holdings (Bøe et al. 2019).

Afforestation as a climate mitigation measure has been extensively criticised by environmental NGOs as well as scientists, due to concerns relating to the negative effects on biodiversity by planning to use the Sitka spruce (*Picea sitchensis*), known to be an alien and invasive species (Backman and Mårald 2016). Concerns relating to impacts on cultural landscapes have also been raised, and that utilising these areas for forestry would constitute an irreversible regrowth on areas that could be used for food production, pastures, tourism and recreation. These debates made the Parliament request changes to the scheme, and in the national budget allocations for 2015 four additional criteria were added to the pilot phase: (i) the use of native Norwegian tree species (most commonly the main commercial tree species in Norway, Norway spruce, *Picea abies*, (ii) planting should take place on open areas and areas in early regrowth state, (iii) afforestation should only be on areas with high production potential and where there is a low expected change in the albedo effect (estimated in Nordland county by comparing global radiation data with maps for duration of snow cover, (iv) planting should be done on areas that are unimportant for biodiversity, recreational interests, cultural heritage or cultural landscapes (Haugland et al. 2015; Bøe et al. 2019).

In the environmental criteria developed for this initiative, it is stated that afforestation on new areas can impact environmental values, such as water quality (Haugland et al. 2013). However, beyond mentioning that forests have potential impacts on water flow in a watershed, only terrestrial environmental criteria are considered in detail. Water-related concerns appear neither in discussions concerning the potential benefits nor in those concerned with problems associated with afforestation. Another reason for why water quality is not taken into account in the development of environmental criteria might be that there is not sufficient relevant baseline data for Norway. There are only a few early studies addressing the potential effects of forest management on water quality (e.g. Haveraaen 1981), and some more recent ones focusing on sea-salt episodes and acidification (Larssen and Holme 2006) and mobilisation of mercury (e.g. de Wit et al. 2014). Most studies on forest management effects have not monitored surface waters and much focus has been on the effects on soil water. With the relatively low number of Norwegian studies, it is more difficult to determinate the potential impacts related to water quality as the Norwegian conditions may differ from other Nordic countries. Moreover, any effect will depend on local conditions as there are considerable differences within the country.

At a general level, afforestation can, however, have significant regional and stand-level consequences for soil and surface water acidification. Forested land generally receives higher amounts of atmospheric deposition of acidifying substances, also called the forest-filter effect (Mayer and Ulrich 1977). In addition, forest growth in itself has an acidifying effect (Tamm and Hallbäcken 1988), due to hydrogen ions replacing base cations taken up by trees. Potential acidification appears not to have been considered in the afforestation scheme, except in relation to change of tree species to Norway spruce (Haugland et al. 2013).

From a Norwegian perspective, the proposal to afforest large areas of coastal land may result in a significant increase in sea-salt related acidification events. In Norway, already forested areas receive about 10% more sulphate deposition and 18% more inorganic N deposition compared to open areas, in what is named “forest-filter” effects (De Schrijver et al. 2007). Excessive deposition of sea salt can result in pronounced short-term depression of pH in surface waters due to cation exchange processes in the soil (Wright et al. 1988; Hindar et al. 1995). Afforestation in Norway can have substantially negative effects on surface waters with regard to mercury, base cations (calcium, magnesium, potassium and sodium), dissolved organic carbon (DOC) and nitrogen (Larssen and Holme 2006; Berthrong et al. 2009). These effects will occur over a long temporal scale. Positive effects of afforestation in a 100-year perspective are likely with increased carbon sequestration and greenhouse gas (GHG) reductions (Futter et al. 2019).

Afforestation is, however, a measure that falls within existing forestry regulations and standards, including requirements for buffer zones. The typical buffer zone along rivers and waterways according to the Norwegian PEFC standard is 10–15 m, while some conditions warrant up to 30 m, although there are exceptions allowing for narrower zones. The standard also state that ground preparation before planting should not be conducted in areas set aside as buffer zones or within 5 m of existing streams with a yearly discharge. In addition, there is also a possibility that increased terrain transport leading to erosion and runoff to rivers and streams has immediate consequence for water quality, if best cutting practices are not adopted.

From our assessment of this scheme, the extent to which water is safeguarded rests on whether the environmental criteria are complied with and the extent to which the forest industry’s own standards (PEFC) with regard to ground preparation, planting and cutting and water considerations are adhered to. However, while the potential effects from afforestation on water quality might be insignificant in the short run, there is less certainty with regard to the long-term effect of such a measure.

### Increased stocking density of existing forests

Increased stocking density of existing forest areas was introduced as a support scheme for climate mitigation in 2016. The initiative seeks to contribute to increased capture of carbon by increasing the production capacity of existing forests after harvesting, either through planting with higher densities or supplemental planting, in a context where stocking density in Norway is often below the optimal level (Søgaard et al. 2015). For the years 2017–2019, 80% of costs for planting of up to 500 plants per ha was reimbursed to forest owners with forest areas that are beyond a minimum plant density threshold depending on “site index” (NAA 2019).

Although presented as a climate-policy measure, increased stocking density as specified by the Norwegian Agriculture Agency, is part of the ordinary silviculture activities, and does not imply changes to how forest areas are managed (NAA 2019). This measure therefore falls under the forest sector’s existing regulations, so that environmental values are also here safeguarded with reference to the national Regulation on Sustainable Forestry (LMD 2006) and the forest industry’s PEFC standard. According to the Norwegian PEFC standard environmental values are to be registered before harvesting, important environmental values and biotopes are to be protected, buffer areas to water bodies are not to be planted, and general outdoor and use interests of the general public are to be heeded.

In addition, it is a requirement that any planting supported through this initiative is mapped accordingly, to ensure that regulations are followed, and further so that control and evaluation can be carried out. The Agency does, however, acknowledge that control of stocking density is difficult and must be based on discretion. We consider that this makes it unlikely that sanctions against forest owners that do not comply with the set guidelines are implemented.

The support scheme has, however, only to a limited extent been utilised by forest owners. This might be a result of complicated procedures for getting support, and that the benefits for the forest owners have not been clearly communicated, as too high plant density also comes with certain risks. When trees are planted at too high densities, self-thinning often occurs due to increased competition for light, water and nutrients (Futter et al. 2019).

However, due to these set environmental criteria and the limited reception, increased stocking density of forest plantations is unlikely to have a substantial effect on water quality and quantity in Norway in the present context.

### Forest fertilisation

Of the three climate mitigation measures for the forestry sector that we assess here, forest fertilisation has had the most popular reception. In the Nordic context, nitrogen (N) fertilisation is commonly used 5-10 years before felling in moderately N deficient forests so as to increase the biomass (Rytter et al. 2016). The fertilisation supported through this scheme is the application of 150 kg of nitrogen per ha 10 years before harvesting (NAA 2016). With this initiative, Norway saw a remarkable increase in fertilised forest areas, from 700 ha nationally in 2015, to 8 379 ha, 9104 ha, and 5648 ha respectively for the years 2016, 2017 and 2018 (SSB 2017, 2019). This is not unprecedented as the Norwegian forestry sector also had periods of high levels of nitrogen fertilisation in earlier times. The 2016 level has, however, not been reached since 1967. Most of this fertilisation (~ 70%) took place in Hedmark in the south-eastern part of Norway, a county known to be the stronghold of forestry.

Forest fertilisation is, and has been, a contested practice (Lindkvist et al. 2011). The main goal of fertilisation is increased production of tree biomass, but through the addition of nutrients fertilisation also has several potential direct and indirect effects, as fertilisation may impact on biodiversity through changes in vegetation and species composition (Strengbom and Nordin 2008; Hedwall et al. 2010, 2013; Sullivan 2018); it may cause shifts in microarthropod communities in the soil (Lindberg and Persson 2004), and can lead to changes in GHG dynamics (Metcalfe et al. 2013). Laudon et al. (2011) points to the potential consequences that fertilisation may have on water quality and the ecology of water bodies. The magnitude and scale of these effects all depend on the application scheme chosen, i.e. the amount of nitrogen added at what time during the growing season. Also, while forest fertilisation might not have noticeable environmental effects immediately, there can be substantial effects on surface water quality upon forest harvesting, depending on which forest harvest method is used (stem-only, whole tree-harvest, light/heavy machinery, etc.) (Futter et al. 2019).

Studies have shown that nitrogen fertilisation leads to detectable short-term increases in soil solution N concentrations (Clarke et al. 2018b), and can also increase N concentrations in streams draining fertilised areas (Laudon et al. 2011; Haugland et al. 2015). The increased N can affect surface water acidification and studies have identified changes in aquatic plant community composition, with a shift towards more N tolerant species (Haugland et al. 2015). However, given the high demand for N in most Nordic forest surface waters, water quality effects are hard to detect even a few hundred meters downstream of fertilised sites (Schelker et al. 2016). Fertilisation with wood ash is currently not allowed in Norway (Regulation on Fertilisers of Organic Origin), but a field experiment has shown no clear short-term effects of wood ash spreading in forests on soil solution chemistry (Clarke et al. 2018b).

In the white paper of 2012, in which forest fertilisation was proposed as a climate mitigation measure, it is stated that the initiative should come with a set of environmental criteria (KLD 2012). These criteria are stated in a joint report from the Norwegian Agricultural Agency and the Norwegian Environment Agency (Haugland et al. 2014). Based on the potential risk that fertilisation might pose to water bodies that are or have been prone to acidification from long-range atmospheric pollution, a protective zone was established for the coastal regions in southern and southwestern parts of Norway. The assessment of environmental criteria sets an upper limit for the 5-year pilot period of fertilisation of 2 500 ha of forests within this zone. During the first year of the pilot, 1 200 ha were fertilised within the protective zone, which is almost half of the total allocation for the trial period. For 2017, another 900 ha of forests were fertilised within the zone, which left some 300 ha for the rest of the trial period. No such restrictions apply to the area outside of this zone, and the extent of the measure is here limited by the annual allocation of funds over the national budget.

However, for fertilisation of forests, both inside and outside of this zone, regulations apply on which areas are to be fertilised (cutting class and vegetation class) and what buffer zones should be adhered to. The Norwegian PEFC standard requires a fertiliser-free zone of 25 metres around lakes, rivers and streams, to minimise nutrient loss and leakage. Mapping has a crucial role for the fertilisation scheme and how it safeguards environmental values. During implementation, environmental values such as surface waters and sensitive or protected nature types are considered through official mapping tools. After validation by the forestry cooperative—sometimes including field visits—these same maps are used for the application of fertiliser by helicopter. These operations produce a track-log that is further presented to the local and national authorities for checking that the requirements are complied with. The municipalities are formally responsible for receiving the applications for reimbursement, and should make sure that necessary documentation is presented, that the operation is mapped, that necessary environmental considerations are taken, and that areas that should not be fertilised are not. That the scheme relies so heavily on maps and mapped datasets, digitalised GPS and fertilising mechanics is key for the way in which the scheme is understood as well as in line with set environmental criteria. While the municipalities have the formal responsibility for ensuring that the environmental criteria are complied with, in practice, however, this is to a large extent left to the forest cooperatives, suggesting a level of uncertainty regarding how the municipalities carry out their responsibilities vested in the Forestry Act.

Hedwall et al. (2014) argue in their analysis of constraints and opportunities for intensifying forestry through fertilisation in northern boreal forests that fertilisation at the moderate scale—comparable to current practice in Norway—would have only small and temporary effect on the environment, but would generate a high rate of return for forest owners. This is also reflective of how the forest fertilisation initiative is perceived by some key actors in this complex, as a *win*–*win* situation in terms of economic benefits to the forest owners and climate gains.

As the fertilisation operations to a large degree rest on these environmental criteria set by the authorities and the standards for sustainable forestry, and in practice ensured by the use of official maps indicating sensitive environmental values, as well as the automated operation of application of fertiliser in line with these criteria and maps, we consider the risks for impacts on surface water from the forest fertilisation *at the point of initiation* to be minimal. This is less certain for the longer term.

## Discussion

Our findings suggest that in the Norwegian context, the legal framework for sustainable forestry, supplemented by the PEFC standard, provides several important safeguards to minimise the effects of the climate mitigation measures in forestry on water resources. Although these activities may have immediate impacts on surface water, e.g. through leaching of nitrogen or through ground preparation, these problems are not likely to occur if regulations and sector standards are complied with. Common for the three initiatives is that they are not novel types of activities, but rather entail increased support to measures already applied in the forestry sector, although the scale of ambitions for both afforestation and fertilisation represents a shift. These same regulations and industry standards also apply at the point of harvesting, but for this last stage of the production cycle, we observe that there is more uncertainty for all the three measures.

The common practice in Norway at present is felling through clear-cutting. This entails stem-only harvesting, as opposed to whole or complete tree harvesting more commonly practised in Sweden and Finland (Futter et al. 2019). In Sweden, intensification in the forestry sector—including increased use of forest fertilisation—has been brought on by increased demands for forest produce and biomass, e.g. in relation to production of bioenergy (Hedwall et al. 2014). In Norway, a value chain for forest biomass for biofuel production has not developed in the same way. One reason might be the difference in the countries’ energy balance; that Norway’s high supply of and reliance on hydropower has left biofuels a less attractive avenue (Scarlat et al. 2011; Forbord et al. 2012; Cavicchi 2018). Another reason might be competition from a more available forest biomass markets in neighbouring Sweden (Cavicchi 2018). While Norwegian authorities in 2008 set goals for doubling the bioenergy production by 2020, this has not materialised. A support scheme for increased outtake of biomass from forests to enhance the value chain was initiated in 2010. Although this was well received by the forest industry it was, however, discontinued after a few years due to little interest and insufficient economic incentives for the forest owner.

This lack of value chains for forest biomass is one important explanation for why the forest industry in Norway has not reached the same levels of intensification as its Nordic counterparts. If the limited value chains are the limiting factor for further intensification of the Norwegian forestry sector, then this might also be what is keeping the impact on surface waters from intensification measures at a minimal level.

In late 2017 plans were launched for building a pilot plant for converting forest biomass to biofuels in Hurum, in south-eastern Norway. When the pilot plant is completed in 2021 it will run a 2-year trial before deciding whether a full-scale biofuel plant will be built (NTB 2017). If so, the ambitions are to produce 1.5 billion litres of biofuels annually, based on non-timber forest biomass such as branches and needles or lower-grade timber. Although this initiative might show the way toward new ways of integrating the forestry sector with the emerging bioeconomy, it remains to be seen whether forest owners will respond to these possible changes in demands for forest produce and forest biomass, and whether harvesting practices in Norway will change. Should a further intensification of the Norwegian forestry sector occur, this might therefore also have bearings on how well suited the legal framework, and the industry’s standards are at safeguarding surface waters.

## Concluding remarks

We have conducted a review of the impact on surface water of three climate mitigation measures introduced to the Norwegian forestry sector; afforestation on new areas, increased stocking density, and forest nitrogen fertilisation. Overall, there is a challenge in separating the impacts of the climate mitigation measures in forestry from the impact of the forestry industry in general. It is also important to emphasise that the measures are not novel in the Norwegian context, but that the facilitation of these measures and the new support schemes introduced suggest an intensification of the existing forest industry.

We have found that several safeguards are in place to minimise the direct effects of forestry measures on water resources. These regulations and guidelines are, however, for the most part geared to safeguard water and environmental values at the point of initiation of these measures, for instance, regarding which areas are suitable for intensification, be it afforestation or fertilisation. There is, however, a large degree of uncertainty as to the long-term effect of all the three measures that have been assessed, but also as to how changing dynamics in the forest industry and adaptations to emerging markets might alter the way the current regulations fit the Norwegian forestry context.

## Footnotes

1 The official estimates of forest area certified under PEFC was reduced from 9100 000 ha (2000–2015) to 7380 750 ha in 2016. This does not reflect a change in certified forest land, but rather that the initial estimates were exaggerated (pers. com. PEFC Norway 21.12.2017).
